# Prenatal Diagnosis of Paternal Uniparental Isodisomy 14 Arising From the Transfer of a Mosaic Monosomy 14 Embryo

**DOI:** 10.7759/cureus.97452

**Published:** 2025-11-21

**Authors:** Josephine Minick, Michelle Lende

**Affiliations:** 1 Department of Obstetrics and Gynecology, Division of Maternal-Fetal Medicine, Baylor College of Medicine, Christus Children's, San Antonio, USA

**Keywords:** genetic counseling, imprinting disorders, mosaic embryo transfer, preimplantation genetic testing, uniparental chromosome 14 disomy

## Abstract

The practice of transferring PGT-A (preimplantation genetic testing for aneuploidy) “mosaic” embryos has become increasingly common, yet there is limited data on the genetic outcomes of these transfers. We herein present a case of fetal paternal uniparental isodisomy of chromosome 14 arising from an embryo categorized as high-level mosaic for monosomy 14. To our knowledge, this case represents the first reported case of uniparental isodisomy following the transfer of a mosaic embryo. This case demonstrates the clinical risk of uniparental disomy after mosaic embryo transfer, adding to a growing body of evidence. This case also emphasizes the need for clear guidelines regarding prenatal diagnostic testing following the transfer of mosaic embryos, and highlights the essential role of prenatal genetics specialists in providing targeted counseling and care for these patients.

## Introduction

Aneuploidy is a leading cause of implantation failure, pregnancy loss, and congenital disorders in infants [1]. For individuals pursuing in vitro fertilization (IVF), preimplantation genetic testing for aneuploidy (PGT-A) is offered to increase the odds of a successful transfer and to decrease the risk of aneuploidy. PGT-A is typically performed through a blastocyst biopsy. At this stage, the embryo comprises two major cell lines: the inner cell mass and the trophectoderm. With a blastocyst biopsy, a hole is drilled in the zona pellucida on day 3; the embryo is then cultured to the blastocyst stage (days 5-6). This allows for a sample of 3-10 trophectoderm cells to herniate through the hole, which are subsequently sampled for testing. Molecular genetic techniques are now used to analyze all 23 pairs of chromosomes via next-generation sequencing. PGT-A is considered a screening modality, as the chromosomal composition of the trophectoderm may not always be consistent with the chromosomal composition of the inner cell mass.

Importantly, a portion of embryos screened on PGT-A will have copy number variation that is not consistent with aneuploidy or euploidy. These embryos are deemed “mosaic”, and can be further classified as high- or low-level mosaic based on the estimated proportion of cells with an abnormal chromosome number. The vast majority of clinics report that 5%-15% of embryos tested on PGT-A are classified as “mosaic”, although specific percentages vary based on the egg contributor's age and the performing laboratory [2].

While the developmental potential of mosaic embryos is still a matter of debate, guidelines and practice have shifted dramatically within the last several years. Historically, many mosaic embryos were not considered for transfer due to concerns for chromosomally abnormal pregnancies and low implantation rates. In 2021, the Preimplantation Genetic Diagnosis International Society (PGDIS) issued guidelines stating that the transfer of mosaic embryos appears to be a relatively safe option for couples, with low risk of negative outcomes [2]. A committee opinion in 2023 from the American Society for Reproductive Medicine (ASRM) had similar guidance [3]. For couples without euploid embryos to transfer and with barriers to additional cycles of IVF, transfer of a mosaic embryo may be an appealing reproductive option. Yet there is still relatively limited data on the long-term genetic outcomes of mosaic embryos following transfer. Larger studies on this topic have focused on outcomes such as rates of positive pregnancy tests, miscarriage, and live birth, which are important metrics, but not necessarily representative of genetic outcomes. Within these studies, genetic evaluation of the pregnancy/infant was often not completed. Furthermore, even the longer term studies did not extend beyond the third year of life [1,4-7]. This is especially important since cases involving mosaic aneuploidies, copy number variants (i.e., “segmental” aneuploidies), or imprinting Kagami-Ogata SyndromeKagami-Ogata Syndromedisorders may be more subtle than common aneuploidies, and therefore may present well after the neonatal period.

While the most obvious genetic concern with the transfer of a mosaic embryo is persistence of aneuploidy in the fetal and/or placental lines, an additional risk is spontaneous resolution of mosaicism (monosomy or trisomy rescue) resulting in uniparental disomy. Uniparental disomy is the inheritance of both copies of a chromosome from a single parent versus the normal pattern of biparental inheritance. Uniparental disomy can result in imprinting disorders - genetic conditions caused by improper expression of a gene based on the parent of origin.

Uniparental disomy can be further described as being iso-disomy (identical copies of the chromosome from one parent) or hetero-disomy (different copies of the chromosome from one parent). This distinction is important because uniparental isodisomy increases the risk for an individual to be affected by a recessive genetic condition for which only one parent is a carrier.

This case focuses on chromosome 14. Both maternal and paternal uniparental disomy for chromosome 14 (UPD14) are associated with well-characterized imprinting disorders [8]. Maternal UPD14 is associated with Temple syndrome (MIM 616222), which is characterized by congenital hypotonia, motor delay, precocious puberty, prenatal and postnatal growth restriction, hyperextensible joints, and mild intellectual disability. Paternal UPD14 is associated with Kagami-Ogata syndrome (MIM 608149). Prenatal features of Kagami-Ogata syndrome include polyhydramnios, macrosomia, placentomegaly, and abdominal/thoracic abnormalities (thoracic dysplasia, omphalocele, diastasis recti, inguinal hernias). Additional thoracic abnormalities include a small, bell-shaped thorax, “coat-hanger” ribs, a narrow chest wall, and cardiac anomalies. These thoracic anomalies can lead to respiratory distress, feeding difficulties, and postnatal growth restriction. Dysmorphic facial features associated with paternal UPD14 include frontal bossing, depressed nasal bridge, hairy forehead, anteverted nares, micrognathia, and/or short neck. Developmental findings include hypotonia, speech and/or motor delays, and normal to mildly impaired intellectual development.

Herein, we present a case of paternal uniparental isodisomy for chromosome 14 in a pregnancy resulting from the transfer of an embryo with high levels of mosaicism for monosomy 14.

## Case presentation

A 44-year-old G2P0010 female was referred for genetic counseling and consultation by a maternal-fetal medicine (MFM) specialist at a gestational age of 21 weeks, 5 days following abnormal microarray results on amniocentesis. The patient was referred to our clinic following this abnormal result as her initial MFM providers were unable to provide comprehensive counseling on the implications of this finding in the absence of an in-house genetic counselor.

The patient and her husband had undergone several cycles of oocyte retrieval with IVF and elected to pursue PGT-A on the resulting embryos. The pregnancy in question resulted from the transfer of a single frozen five-day blastocyst with high-level (40%-80%) mosaicism for monosomy 14. The couple had received pre-transfer counseling on all of their embryos from a genetic counselor at the laboratory that performed their PGT-A.

Non-invasive prenatal screening was low risk for trisomy 13, 18, 21, and sex chromosome aneuploidies. Increased nuchal thickness was identified in the first trimester, and the sonographic estimated fetal weight was at the 88th percentile at 20 weeks, 4 days of gestational age.

The patient underwent amniocentesis at 16 weeks, 5 days gestation due to the mosaicism reported on PGT-A. Maternal cell contamination studies were negative. Fluorescent in situ hybridization for chromosomes 13, 18, 21, X and Y was normal. Chromosome analysis was consistent with a typical male karyotype (46, XY). Single-nucleotide polymorphism microarray on cultured amniocytes showed normal dosage but whole chromosome 14 homozygosity, consistent with a diagnosis of uniparental isodisomy 14. No mosaicism was noted on karyotype or microarray.

**Figure 1:**
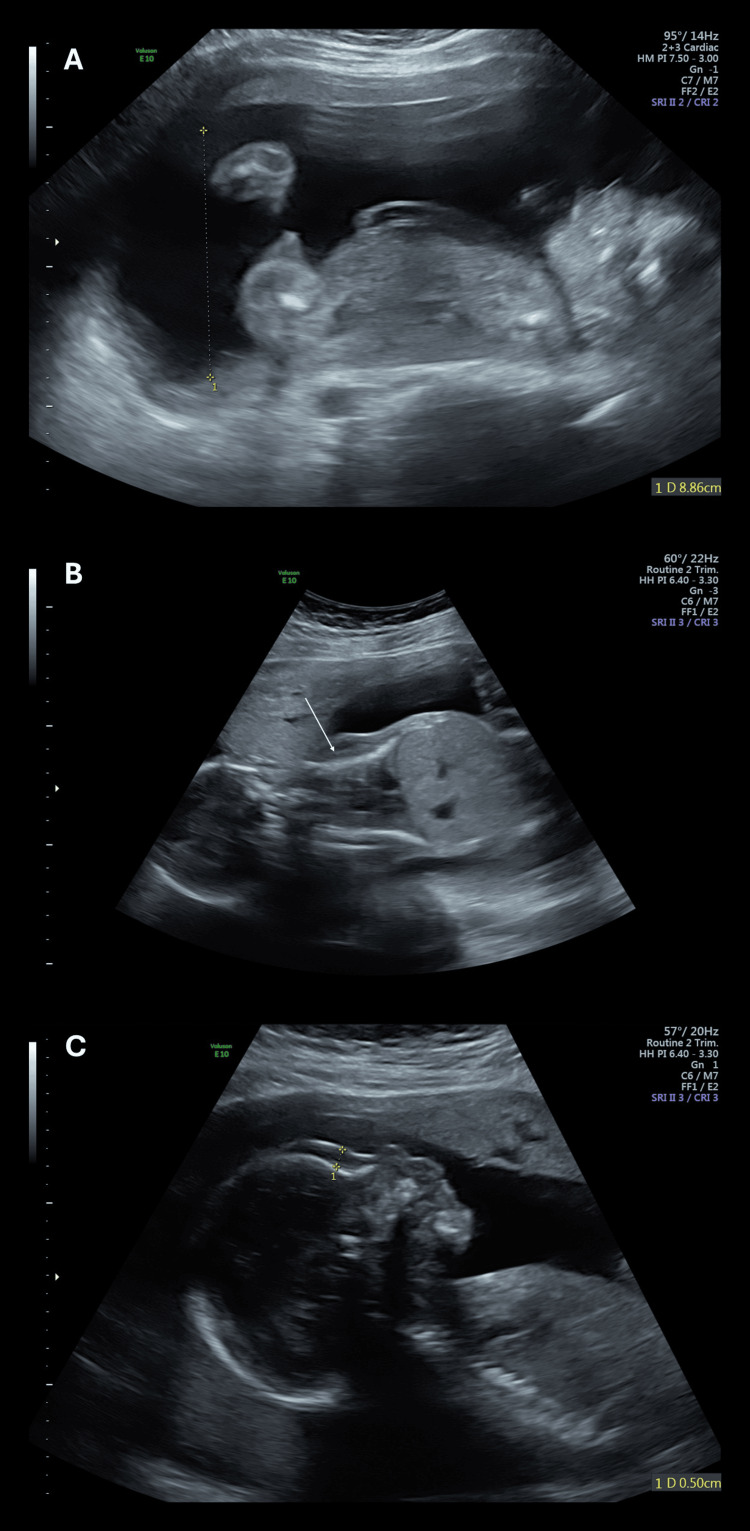
Ultrasound findings suggestive of paternal uniparental isodisomy 14 (A) Maximum vertical pocket (MVP) measurement indicative of polyhydramnios, (B) subjectively narrow chest, (C) abnormal facial profile with thickened skin.

A repeat anatomy scan at 21 weeks, 5 days gestation focused on features associated with UPD14. Features identified on this targeted scan included polyhydramnios, a subjectively small thorax, fetal edema on the face and head, and abnormally positioned hands with closed fists (Figure 1). The fetus continued to measure large at the 93rd percentile for gestational age. Together, these sonographic findings were consistent with paternal UPD14.

Following this anatomy scan, paternal UPD14 was confirmed on microsatellite testing on DNA from amniocytes. Based on the severe prognosis of paternal UPD14, the patient underwent a dilation and evacuation at 23 weeks of gestational age. The procedure was reportedly uncomplicated. No further genetic evaluation was completed on products of conception.

## Discussion

This case report represents an important contribution to the growing literature surrounding genetic outcomes of mosaic embryos. There are a few case studies in the medical literature demonstrating the persistence of embryonic mosaicism in placental or fetal tissues. Greco et al. in 2023 reported a case of true fetal mosaicism for 1p36 following transfer of an embryo with segmental mosaicism for this region, and another of fetal mosaicism for trisomy 21 following transfer of an embryo with a complex mosaic pattern [9]. Kahraman et al. reported a case of low-level trisomy 2 resulting from the transfer of an embryo with monosomy 2 mosaicism [10]. Mounts et al. presented a case where an apparently non-mosaic 15q11.2-q13.1 duplication was identified in an infant conceived from an embryo with mosaicism for the duplication [11]. Schlade-Bartusiak et al. presented a case of maternal uniparental disomy 15 and partial trisomy 15 in an infant conceived following transfer of an embryo with high-level mosaic trisomy 15 and high-level mosaic deletion on chromosome 20 (mos(del(20)(q11.23-qter)) [12]. Viotti et al. (2023) reported three cases of mosaicism persisting to the prenatal stage following transfer of mosaic embryos [13].

This case demonstrates an apparently non-mosaic fetal imprinting disorder arising from embryonic mosaicism. Findings on PGT-A and genetic testing on amniocentesis suggest that a series of segregation errors occurred beginning in maternal meiosis. The fertilized egg likely had monosomy 14 resulting from maternal meiotic nondisjunction. Before the fifth day of development, a further mitotic error (“monosomy rescue”) must have occurred, resulting in a mix of aneuploid (-14) and euploid cells. This would have caused the mosaicism for monosomy 14 that was detected on PGT-A. Importantly, both copies of the 14th chromosome in these cells would be genetically identical and paternally imprinted.

Notably, no mosaicism for monosomy 14 was identified on karyotype or microarray. This could also indicate that the monosomic 14 cell lines were outcompeted or died off. Alternatively, this could indicate that monosomy rescue occurred early enough in development that the mosaicism was limited to the trophectoderm tissue. Low-level mosaicism for monosomy 14 cannot be ruled out due to the DNA source (microarray was completed on cultured amniocytes), limited cell counts on chromosome analysis (15 metaphases counted, 5 analyzed, 3 karyotyped), or the fact that additional fetal tissues were not sampled.

This case illustrates the need for additional guidance from professional bodies regarding the prioritization of embryos for transfer. While previous iterations of PGDIS statements included specific guidance regarding suggested prioritization based on PGT-A results, this was removed in the 2021 iteration [2]. In this case, the couple had another embryo available for transfer with high-level mosaicism for monosomy 2. While all mosaic embryos carry some genetic risk, this embryo would likely have had relatively less genetic risk due to the lack of association with imprinting disorders. Although other factors, such as embryo morphology, may have influenced the decision regarding which embryo to transfer, it is unclear if the risk from imprinting disorders was duly considered when selecting the embryo for transfer.

The case also reflects the need for personalization of counseling and genetic testing plans for embryos with mosaicism on PGT-A. While the patient had preconception genetic counseling at the PGT-A laboratory, it did not translate into changes in prenatal care. This is evident by the patient's recall that microarray was treated as an add-on “supplemental test” following the normal karyotype on their amniocentesis sample. The counseling and testing strategy utilized in this instance was not tailored to the specific genetic risks identified by PGT-A for this embryo. This caused significant delays in diagnosis, increased psychosocial stress on the family, and limited interpretation of some results (i.e., insufficient cell counts on karyotype, utilizing cultured vs. direct amniocytes for microarray, not ordering microsatellite testing after UPD was identified). Because of the increased risks associated with mosaic embryos, these patients may benefit greatly from thorough, personalized genetic counseling regarding the possible genetic outcomes of their embryo(s), options for genetic screening and diagnosis in pregnancy, and recommended prenatal genetic test(s) specific to their PGT-A results. This is in alignment with recommendations by ASRM [3].

In addition, uniparental isodisomy of any chromosome increases the risk for a fetus to be affected by a recessive condition for which only one parent is a carrier. While this is not the primary risk associated with the transfer of a mosaic embryo, parental carrier status should be considered when considering the transfer of a mosaic embryo. While reproductive risk is generally thought to be low for most autosomal recessive conditions for which only one parent is a carrier, this may not be the case for mosaic embryos at increased risk of uniparental isodisomy. For this couple, carrier screening had been completed for the pregnant patient, but no testing was thought to be necessary for the father of the baby. This is a reasonable course of practice when biparental inheritance is assumed, but may not be appropriate in cases with increased risk of uniparental disomy.

## Conclusions

In conclusion, this case represents a unique proof of concept regarding the genetic risks that exist following transfer of a known mosaic embryo. In this case, the genetic risk for this pregnancy was not the persistence of the monosomic line, but the consequences of monosomic rescue resulting in an imprinting disorder. Additional research on how often diagnostic genetic testing is pursued following mosaic embryo transfer, and how often mosaicism identified on PGT-A results in clinically significant genetic consequences, is needed.

This case underscores the pivotal role of genetics professionals (such as board-certified genetic counselors) in managing mosaic embryo transfers. In the pre-transfer space, genetic counselors can provide detailed preimplantation genetic counseling to the patients and give input to the patient's physicians regarding genetic risks that may influence embryo selection. Post-transfer, genetic counselors can support patients and physicians with interpretation of the unique genetic risks, recommendations regarding prenatal genetic testing, and individualized interpretation of genetic results. Throughout the process of a mosaic embryo transfer, specialized genetics knowledge and counseling are necessary to allow informed decision-making and provide the best quality of care.
